# Supplementary material for: From sequence to enzyme mechanism using multi-label machine learning
Source: BMC Bioinformatics. 2014 May 19;15:150. doi: 10.1186/1471-2105-15-150 (PMC4229970; doi:10.1186/1471-2105-15-150)
Supplement: Additional file 2 — Java code of ml2db. Additional file ml2db_code.tar.gz contains the Java source code to run the multi-label machine learning experiments and save the results to database. The code’s Javadoc is included. [file 1471-2105-15-150-S2.zip › additional file 2/ml2db/ml2db/doc/mulan/dimensionalityReduction/Ranker.html]

Ranker


JavaScript is disabled on your browser.


- Overview
- Package
- Class
- Use
- Tree
- Deprecated
- Index
- Help

- Prev Class
- Next Class

- Frames
- No Frames

- All Classes

- Summary:
- Nested |
- Field |
- Constr |
- Method

- Detail:
- Field |
- Constr |
- Method


mulan.dimensionalityReduction

## Class Ranker

- java.lang.Object
- - mulan.dimensionalityReduction.Ranker

- ---

    

  ```
  public class Ranker
  extends java.lang.Object
  ```

  Ranks attributes according to an AttributeEvaluator. It internally uses Weka's
  Ranker, initialized so as to neglect the labels.

  Version:
  :   2012.05.30

  Author:
  :   Grigorios Tsoumakas

- - ### Constructor Summary

    Constructors

    Constructor and Description

    `Ranker()`
  - ### Method Summary

    Methods

    | Modifier and Type | Method and Description |
    | --- | --- |
    | `int[]` | `search(weka.attributeSelection.AttributeEvaluator attributeEval, MultiLabelInstances mlData)` Calls a specified `AttributeEvaluator` to evaluate each feature attribute of specified `MultiLabelInstances` data set, excluding labels. |

    - ### Methods inherited from class java.lang.Object

      `equals, getClass, hashCode, notify, notifyAll, toString, wait, wait, wait`

- - ### Constructor Detail


    - #### Ranker

      ```
      public Ranker()
      ```
  - ### Method Detail


    - #### search

      ```
      public int[] search(weka.attributeSelection.AttributeEvaluator attributeEval,
                 MultiLabelInstances mlData)
                   throws java.lang.Exception
      ```

      Calls a specified `AttributeEvaluator` to evaluate each feature
      attribute of specified `MultiLabelInstances` data set, excluding
      labels. Internally it uses `Ranker`

      Parameters:
      :   `attributeEval` - the attribute evaluator to guide the search
      :   `mlData` - the multi-label instances data set

      Returns:
      :   an array (not necessarily ordered) of selected attribute indexes

      Throws:
      :   `java.lang.Exception` - if an error occur in search


- Overview
- Package
- Class
- Use
- Tree
- Deprecated
- Index
- Help

- Prev Class
- Next Class

- Frames
- No Frames

- All Classes

- Summary:
- Nested |
- Field |
- Constr |
- Method

- Detail:
- Field |
- Constr |
- Method
